# Comparative Transcriptomic Analysis of *Staphylococcus aureus* Reveals the Genes Involved in Survival at Low Temperature

**DOI:** 10.3390/foods11070996

**Published:** 2022-03-29

**Authors:** Biao Suo, Peng Guan, Zijie Dong, Yun Zeng, Shijia Fan, Huiping Fan, Zhongmin Huang, Zhilu Ai

**Affiliations:** 1 College of Food Science and Technology, Henan Agricultural University, Zhengzhou 450002, China; suobiao1982@126.com (B.S.); guanp1996@163.com (P.G.); dongzijie96@163.com (Z.D.); zengyun@muyuanfoods.com (Y.Z.); f1830768908@163.com (S.F.); fanhuiping1972@hotmail.com (H.F.); zmhuang2000@163.com (Z.H.); 2 Key Laboratory of Staple Grain Processing, Ministry of Agriculture and Rural Affairs, Zhengzhou 450002, China; 3 Henan Engineering Laboratory of Quick-Frozen Flour-Rice Food and Prepared Food, Henan Engineering Research Center for Cold-Chain Food, Henan Agricultural University, Zhengzhou 450002, China

**Keywords:** *Staphylococcus aureus*, low temperature, oxidative stress, energy metabolism, cell structure, RNA-seq

## Abstract

In food processing, the temperature is usually reduced to limit bacterial reproduction and maintain food safety. However, *Staphylococcus aureus* can adapt to low temperatures by controlling gene expression and protein activity, although its survival strategies normally vary between different strains. The present study investigated the molecular mechanisms of *S. aureus* with different survival strategies in response to low temperatures (4 °C). The survival curve showed that strain BA-26 was inactivated by 6.0 logCFU/mL after 4 weeks of low-temperature treatment, while strain BB-11 only decreased by 1.8 logCFU/mL. Intracellular nucleic acid leakage, transmission electron microscopy, and confocal laser scanning microscopy analyses revealed better cell membrane integrity of strain BB-11 than that of strain BA-26 after low-temperature treatment. Regarding oxidative stress, the superoxide dismutase activity and the reduced glutathione content in BB-11 were higher than those in BA-26; thus, BB-11 contained less malondialdehyde than BA-26. RNA-seq showed a significantly upregulated expression of the fatty acid biosynthesis in membrane gene (*fabG*) in BB-11 compared with BA-26 because of the damaged cell membrane. Then, catalase (*katA*), reduced glutathione (*grxC*), and peroxidase (*ahpC*) were found to be significantly upregulated in BB-11, leading to an increase in the oxidative stress response, but BA-26-related genes were downregulated. NADH dehydrogenase (*nadE*) and α-glucosidase (*malA*) were upregulated in the cold-tolerant strain BB-11 but were downregulated in the cold-sensitive strain BA-26, suggesting that energy metabolism might play a role in *S. aureus* under low-temperature stress. Furthermore, defense mechanisms, such as those involving *asp23*, *greA,* and *yafY,* played a pivotal role in the response of BB-11 to stress. The study provided a new perspective for understanding the survival mechanism of *S. aureus* at low temperatures.

## 1. Introduction

*Staphylococcus aureus* is a Gram-positive bacterium that infects the human body and produces enterotoxins [1,2]. According to a report by the Centers for Disease Control and Prevention [3,4], approximately 241,000 foodborne diseases are caused by *S. aureus* each year in the United States, and it is the third most prevalent foodborne disease in the world [5], as it is often present in frozen and refrigerated food, such as meat, aquatic products, and ice cream [6].

The cell membrane of bacteria plays an important role in maintaining cell morphology, exchanging substances with the outside world, and preserving the relative stability of the intracellular environment [7]. When the cell membrane is destroyed, the integrity change causes the cellular content to flow out, followed by cell lysis and death [8,9]. Cell membrane damage is caused by a variety of conditions; extreme temperatures will decrease cell membrane fluidity and cause it to solidify and break [10]. When the organism is harmed, it will produce reactive oxygen species (ROS), which attack the polyunsaturated fatty acids in the cell membrane, causing lipid peroxidation to destroy the structure and change the integrity [11,12].

Bacterial survival mechanisms regulate cellular metabolites and proteins through gene expression in response to exposure to different pH levels, temperature, osmotic pressure, and toxic chemicals, thereby maximizing the chance of survival [13,14]. For example, *S. aureus* overexpresses the *csp* family gene to enhance its cold stress response at low temperatures [15]; *S. aureus* undergoes exhibits of amino acids, citric acid, and β-aminoisobutyric acid after cold stress, allowing the bacteria to adapt to long-term low-temperature environments [16].

The important role of RNA makes it the primary factor to analyze for a comprehensive description of the physiological state of cells [17], revealing the association between gene expression and life activities [18]. In recent years, the effect of low-temperature stress on *S. aureus* has been reported, but the object of the analysis is generally a single sample. However, small genetic differences affect stress tolerance in bacteria, and differences between sensitive isolates and tolerant isolates have been identified [19]. Therefore, by comparing strains with different resistance levels, we obtained a comprehensive understanding of which molecular mechanisms played a role in regulating stress resistance [20].

Therefore, the purposes of this study were (1) to explore the differences in the cell membrane integrity of *S. aureus* with different survival strategies at a low temperature and (2) to study the differences in the changes in oxidative stress in *S. aureus* at a low temperature. (3) Based on an RNA-Seq analysis of the differences in transcript levels in *S. aureus* with different survival strategies for exposure to a low temperature, we discovered the role of stress resistance-related genes in this study.

## 2. Materials and Methods

### 2.1. Bacterial Strains and Culture Conditions

The two *S.aureus* strains, BB-11 and BA-26, used in this study, were collected in our lab from meat samples and were confirmed to be *S. aureus* using the Biolog technique [21] and PCR amplification of the *nuc* gene [22]. After homogenizing 25 g meat samples, they were placed in 225 mL of trypticase soy broth supplemented with 0.6% (*w*/*v*) yeast extract (TSB-YE) (Land Bridge Technology Co., Ltd., Beijing, China) and 10% (*w*/*v*) sodium chloride (Fuyu Technology Co., Ltd., Tianjin, China) at 37 °C, 150rpm/min shaker culture for 18h. Then, separation and purification on tryptone soy agar with 0.6% (*w*/*v*) yeast extract (TSA-YE) (Land Bridge Technology Co., Ltd., Beijing, China) plates were repeated three times [23]. The two strains were stored in trypticase soy broth supplemented with 0.6% (*w*/*v*) yeast extract (TSB-YE) and 50% (*v*/*v*) glycerin (Fuyu Technology Co., Ltd., Tianjin, China) at −80 °C until use.

### 2.2. Preparation of Bacterial Suspensions and Cold Treatment

The *S. aureus* was inoculated in TSB-YE and incubated at 37 °C with shaking until the late exponential stage, 25 mL of cell culture medium were collected by centrifugation at 8000 rpm for 5 min at 4 °C, and bacteria were washed three times with phosphate-buffered saline (PBS) (Solarbio Technology Co., Ltd., Beijing, China). Then, the cells were resuspended in an equal volume of PBS to a final concentration of 9.5 logCFU/mL in a 50 mL plastic tube. For low-temperature treatment, the suspension was stored at 4 °C in a refrigerator for subsequent determination.

### 2.3. Viable Cell Enumeration

The total number of colonies was determined weekly for 4 weeks of low-temperature treatment by spreading suitable dilutions of the low-temperature treated cells on TSA-YE plates. Three parallel replicates, one of each sample, were analyzed.

### 2.4. Nucleic Acid Leakage Analysis

After 1 week of low-temperature treatment, 1 mL of the cell suspension was transferred to a 1.5 mL RNase/DNase-free centrifuge tube. The culture was centrifuged at 8000 rpm for 5 min at 4 °C. Then, the centrifuged supernatant was transferred to a new centrifuge tube by pipettor. Using a NanoDrop 2000 spectrophotometer (Thermo Fisher Scientific Co., Ltd., Wilmington, NC, USA) the optical density of the supernatant was measured at 260 nm, the amount of intracellular nucleic acid leakage was calculated [24]. All operations were performed on ice to minimize nucleic acid degradation.

### 2.5. Determination of Microscale Malondialdehyde (MDA) and Reduced Glutathione (GSH) Contents, and Superoxide Dismutase (SOD) Activity

One milliliter each of the two strains of low-temperature treated *S. aureus* suspension was collected into separate centrifuge tubes, centrifuge at 8000 rpm for 5 min at 4 °C, and the supernatant was discarded. The extraction solution was added at a ratio of bacterial number: extraction solution of 1000:1. Then, the bacteria were crushed with a JY99-IIDN ultrasonic device (Xinzhi Biotechnology Co., Ltd., Ningbo, China; ice bath, 200 W, ultrasound 3 s, interval 10 s, and repeat 30 times). The samples were centrifuged at 8000 rpm for 10 min at 4 °C. The supernatant was placed on ice to determine the MDA and GSH contents, as well as the SOD activity using the microscale malondialdehyde (MDA) assay kit, reduced glutathione (GSH) assay kit, and superoxide dismutase (SOD) assay kit (Jiancheng Technology Co., Ltd., Nanjing, China), according to the manufacturer’s recommendations.

### 2.6. Confocal Laser Scanning Microscopy (CLSM) Observation

One milliliter of the late exponential stage of *S. aureus* treated with a low temperature for 1 week was centrifuged at 8000 rpm for 10 min and washed 3 times with sterile PBS. *S. aureus* was incubated with reagents from the Calcein-AM/PI kit at 37 °C for 30 min in the dark prior to CLSM observation [25]. After entering the cell, calcein-AM was hydrolyzed by endogenous esterases in living cells to produce the polar molecule calcein (calcein), which has a strong negative charge and does not penetrate the cell membrane; thus, it is retained in the cell, while calcein emits strong green fluorescence. The nucleic acid red fluorescent dye propidium iodide (PI) does not penetrate the cell membrane of living cells, but only stains dead cells whose cell membrane integrity has been destroyed. Therefore, live cells will emit green fluorescence after staining, while dead cells will emit red fluorescence. According to the manufacturer’s recommendations, a Nikon A1R confocal laser scanning microscope (Nikon, Japan) was used to capture images, and the NIS-Elements Viewer software was used for image analysis.

### 2.7. Transmission Electron Microscopy (TEM) Observation

Referring to the method reported by Suo et al. [26], 1 mL of *S. aureus* after 1 week of low-temperature treatment and 1 mL of the late exponential stage of *S. aureus* bacterial suspensions were washed 3 times with PBS and fixed with 4% (*v*/*v*) glutaraldehyde (Fuyu Co., Ltd., Tianjin, China) for 4 h at 4 °C, then washed 4 times with PBS. The samples were then dehydrated in different gradients of acetone (Tianjin Co., Ltd., Tianjin, China) solutions and embedded in an embedding medium (Epon812, Zhongjing Technology Co., Ltd., Beijing, China) for 4 h. Ultrathin sections (EM UC6, Leica Co., Ltd., Solms, Germany) were stained with uranyl acetate and lead citrate (Fuyu Co., Ltd., Tianjin, China) for 10 min and observed using a JEM-1400 transmission electron microscope (EOL Japan Electronics Co., Ltd., Tokyo, Japan).

### 2.8. RNA-Seq Analysis

Total RNA was extracted from 1 mL of *S. aureus* after 1 week of low-temperature treatment and 1 mL of the late exponential stage of *S. aureus* bacterial suspensions using TRIzon reagent (Kangwei Century Biotechnology Co., Ltd., Nanjing, Jiangsu, China), followed by sequencing, transcriptome assembly, and annotation. After constructing a cDNA library, the raw data (raw data) usually contain a small amount of junction contamination and low-quality reads, which must be filtered and rehybridized. The Bowtie2 alignment software [27] was used to compare the clean reads (reads obtained after filtering were completed) obtained from the sequencing of each sample with the reference *S. aureus* genome (GCF_013307085.1) (https://www.ncbi.nlm.nih.gov/assembly/GCF_013307085.1/ (accessed on 12 July 2021)), and two base mismatches were allowed during the alignment process. Gene expression levels in each sample were analyzed using HTSeq software [28], the expression of each gene was tested for the null hypothesis using a negative binomial distribution statistical model to obtain *p* values for comparison and differentially expressed genes (DEGs) were screened according to a *p* value ≤ 0.05 and FC ≥ 2.

GO enrichment analysis was used to identify the main biological functions performed by DEGs [29]. All the differentially expressed genes obtained above were mapped to the Gene Ontology (GO) database (http://www.geneontology.org/, accessed on 12 July 2021). The Kyoto Encyclopedia of Genes and Genomes (KEGG) (https://www.kegg.jp/, accessed on 12 July 2021) was used to predict the pathways of the DEPs. The pathway annotation information corresponding to the differentially expressed genes was obtained.

### 2.9. Validation of RNA-Seq Results Using qRT–PCR

qRT–PCR was performed to validate the transcript levels identified using RNA-Seq. The primers were designed and validated by Primer-BLAST (https://www.ncbi.nlm.nih.gov/, accessed on 18 October 2021) and synthesized by Shangya Biological Co., Ltd. (Zhengzhou, China); see Table 1 for details. PCR products were amplified and detected using a StepOnePlus instrument (ABI Co., Ltd., Waltham, MA, USA). The following amplification procedure was used: 95 °C, 5 min for the predenaturation step; 95 °C, 10 s for denaturation; 60 °C, 20 s for annealing; 72 °C, 20 s for extension; followed by 40 cycles of denaturation, annealing, and extension. Solubility curves were generated using the default settings of the instrument. The data were quantified using the *2*^−ΔΔ*Ct*^ method [30] and 16S rDNA served as the internal reference gene.

### 2.10. Statistical Analysis

All experiments were performed in three parallel replicates to calculate the mean and error (Excel 2019, Microsoft, Albuquerque, NM, USA), and one-way ANOVA (SPSS, IBM Co., Ltd., Armonk, NY, USA) was used for statistical analyses of the significance of differences at the *p* < 0.05 level. The results were plotted using Origin 8.5 software (OriginLab Co., Ltd., Northampton, MA, USA).

## 3. Results and Discussion

### 3.1. Changes in the Number of Viable Cells and Intracellular Nucleic Acid Leakage of S. aureus

**Figure 1:**
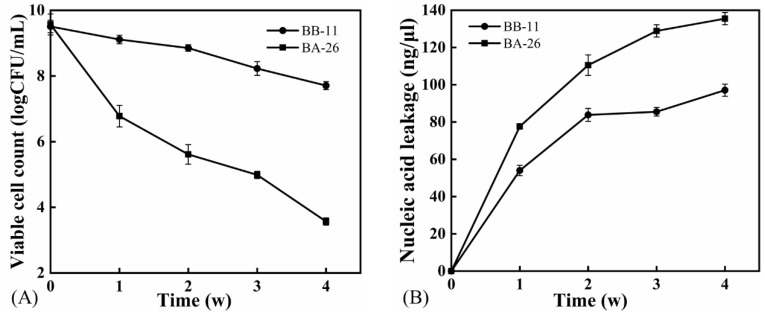
Viable cell number growing on TSA-YE plates (**A**) and intracellular nucleic acid leakage; (**B**) of *S. aureus* after low temperature (4 °C) treatment. Error bars represent standard deviations (*n* = 3).

The number of viable cells of both *S. aureus* strains decreased continuously under low-temperature stress (Figure 1A). As shown in the figure, the number of viable BA-26 cells decreased by 6.0 logCFU/mL after 4 weeks of low-temperature treatment, while only a 1.8 logCFU/mL decrease was observed for *S. aureus* strain BB-11. Based on these results, BB-11 was more resistant to low temperatures than the BA-26 strain.

As shown in Figure 1B, the amount of intracellular material leakage from both strains of *S. aureus* increased continuously during the low-temperature treatment, with the lowest nucleic acid leakage of 97.1 ng/μL observed for BB-11; however, the amount of nucleic acid leakage was 135.5 ng/μL for BA-26, which was 38.4 ng/μL higher than that of BB-11 (*p* < 0.05). Therefore, the cell membrane of *S. aureus* was disrupted and intracellular nucleic acid efflux was increased after low-temperature treatment, while the amount of nucleic acid leakage from BB-11 was less than that from BA-26. Because the BA-26 strain has more apoptosis after long-term low-temperature treatment, it is difficult to carry out biochemical analysis, thus *S. aureus* after 7 days of low-temperature treatment was selected for follow-up experiments.

### 3.2. Oxidative Stress in S. aureus

**Figure 2:**
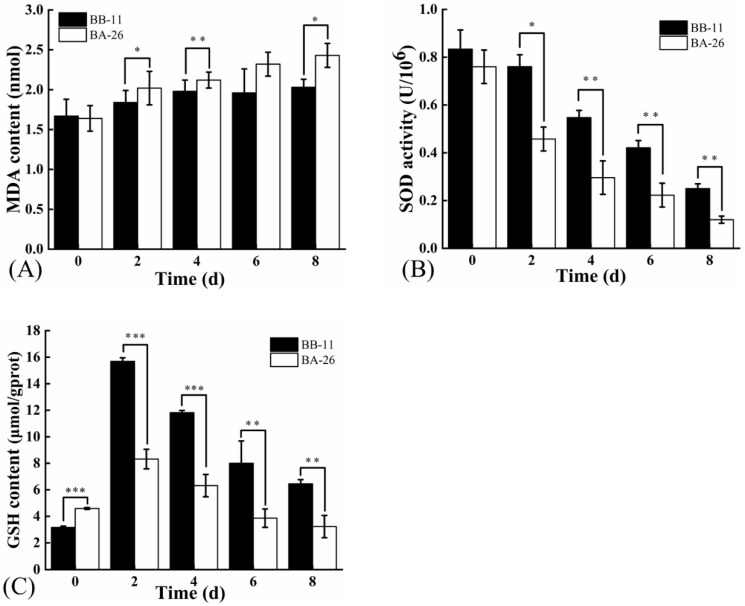
Effect of low temperature (4 °C) on the malondialdehyde (MDA) content (**A**), superoxide dismutase activity (SOD) (**B**) and reduced glutathione (GSH) content (**C**) in *S. aureus.* Error bars represent standard deviations (*n* = 3) (* *p* < 0.05, ** *p* < 0.01, and *** *p* < 0.001).

In Figure 2A, the MDA content in both strains of *S. aureus* increased during the low-temperature treatment, but the MDA content in BB-11 was always lower than that in BA-26. The increasing trend for the MDA content in BB-11 decreased from 4 to 8 days, while the MDA content in BA-26 increased throughout this period, reaching the maximum difference of 0.4 nmol at 8 d. In Figure 2B, the SOD activity of both strains decreased after low-temperature stress, but the SOD activity of BB-11 was higher than BA-26 throughout the 8-day analysis. Changes in the reduced glutathione content in *S. aureus* are shown in Figure 2C. The reduced glutathione content in both strains increased after a short period of low-temperature treatment; however, the content of reduced glutathione in BB-11 was 7.36 μmol/gprot higher than that in BA-26 on the second day (*p* < 0.001). However, the reduced glutathione content decreased with the increasing time of low-temperature treatment, except that the content in BB-11 was always significantly higher than that in BA-26 (*p* < 0.001). Because the SOD activity and reduced glutathione contents were more difficult to measure with the increase in the time of low-temperature treatment, the experimental data are only listed for up to 8 d.

### 3.3. CLSM Observations

**Figure 3:**
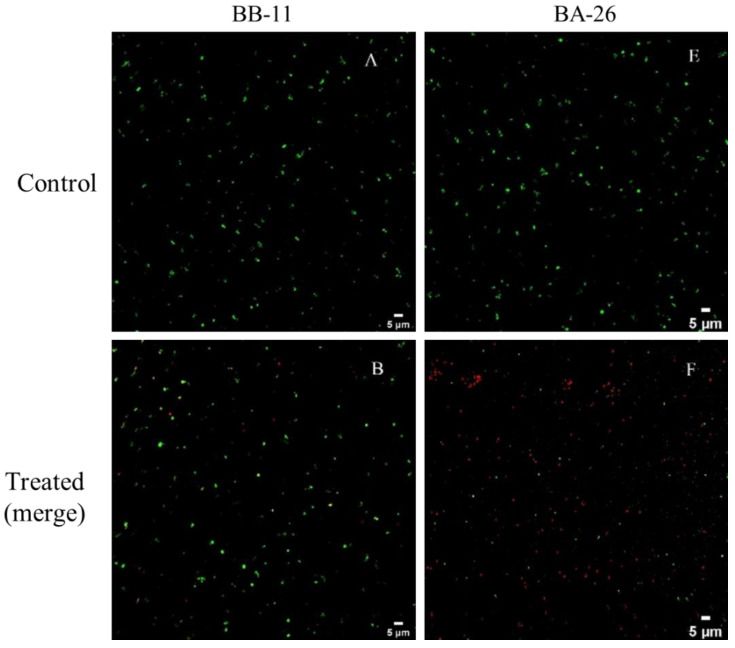

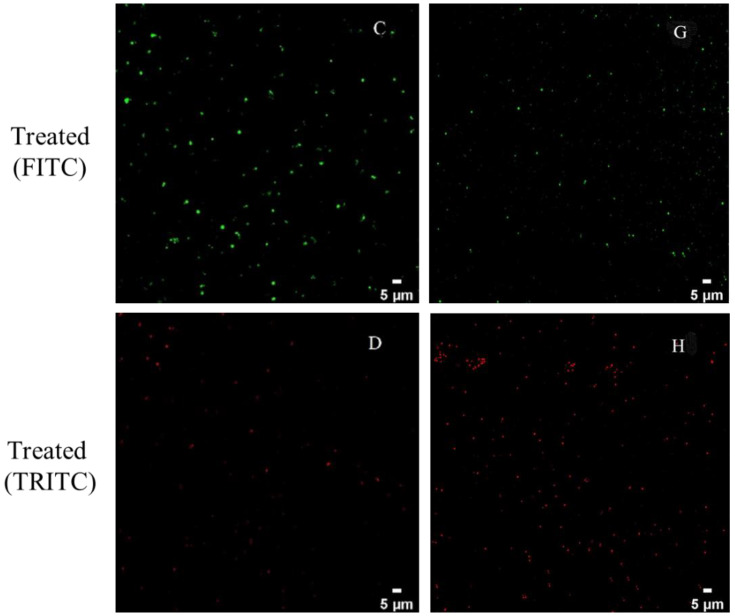
Confocal laser scanning microscopy images of *S. aureus* in the late exponential stage and exposed to low temperature (4 °C). (**A**,**E**): BB-11 and BA-26 without low temperature treatment; (**B**,**F**): merge images with full fluorescence channel of BB-11 and BA-26 after low temperature treatment; (**C**,**G**): green fluorescence channel (FITC) images of BB-11 and BA-26 after low temperature treatment; (**D**,**H**): red fluorescence channel (TRITC) images of BB-11 and BA-26 after low temperature treatment The green fluorescence (stained with calcein-AM) indicates that the cell membranes remain intact and bacteria are considered live, and the red fluorescence (stained with propidium iodide) indicates the disruption of membranes and bacteria are considered dead. Scale bar = 5 μm for each CLSM image.

Using fluorescent dyes to stain the cells, as shown in Figure 3, most *S. aureus* cells in the two control groups were stained green, indicating that the bacteria survived and the cell membranes were intact. However, most BA-26 cells stained red after 1 week of low-temperature treatment and only a small portion was green, indicating that the number of cells with intact membranes was significantly reduced (*p* < 0.05). In contrast, most of the BB-11 cells were still green after 1 week of low-temperature treatment, and only a small portion of the cells was red. Based on these results, BB-11 still retained its cell membrane integrity after low-temperature treatment, and the membranes of only a few cells lost selective permeability and exhibited red fluorescence; the number of dead cells only increased to a lower extent. After BA-26 underwent low-temperature treatment, most of the cell membranes lost selective permeability and a substantial number of dead cells was observed.

### 3.4. TEM Observations

**Figure 4:**
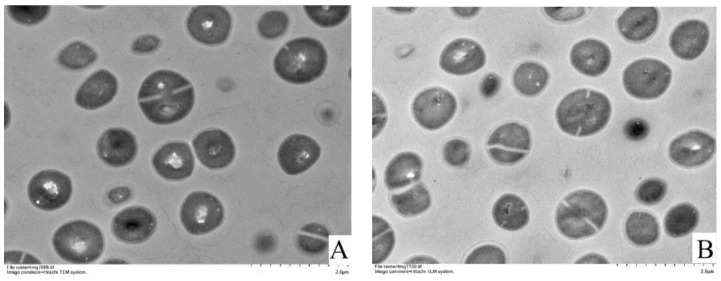

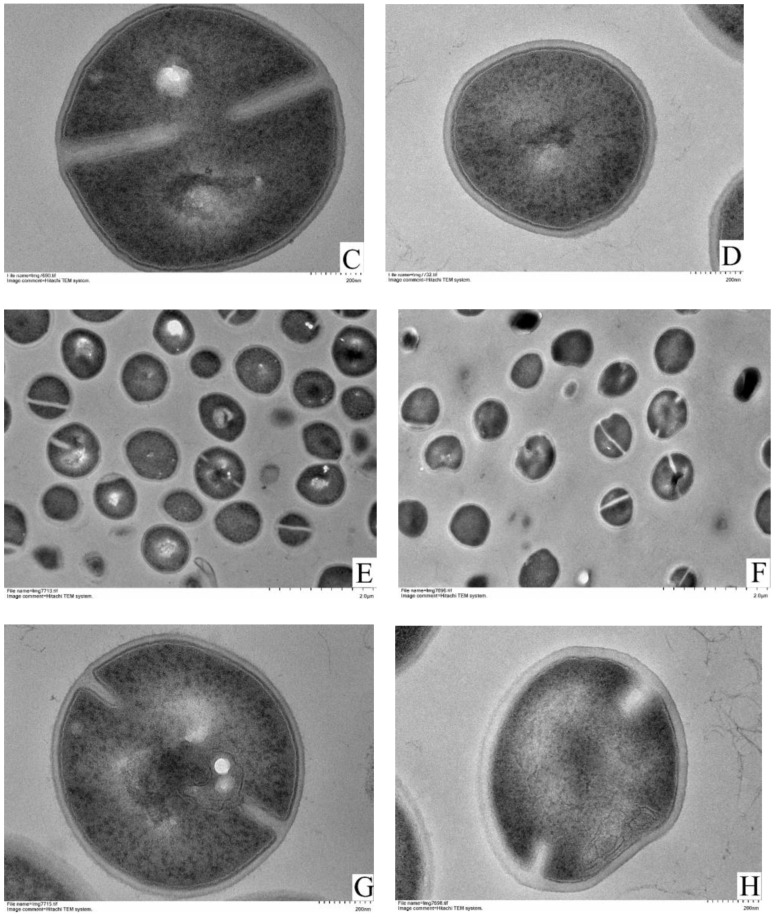
Transmission electron microscopy images of *S. aureus* in the late exponential stage and exposed to a low temperature (4 °C). White arrows indicate the cells with more severe cell membrane wrinkling. (**A**,**C**): The late exponential stage of BB-11; (**B**,**D**): the late exponential stage of BA-26. (**E**,**G**): BB-11 exposed to a low temperature; (**F**,**H**): BA-26 exposed to a low temperature. Scale bar = 2 μm in images shown in (**A**,**B**,**E**,**F**) (30,000× magnification); scale bar = 200 nm in images shown in (**C**,**D**,**G**,**H**) (4000× magnification).

As shown in Figure 4, transmission electron microscopy showed that the cell wall and cell membrane were intact. The membrane integrity of BB-11 cells was not different from that of fresh cells, and only a few cells were wrinkled. When BA-26 was exposed to a low temperature, most of the cell profiles were changed, and the number of cells with a crinkled morphology accounted for 35.71% of all cells in the field of view (white arrows indicate cells with more severe cell membrane wrinkling). Therefore, after low-temperature treatment, the BB-11 cell membrane changed to a lesser extent while the BA-26 cell membrane was more wrinkled in a greater number of cells, consistent with the results of intracellular nucleic acid leakage and confocal laser scanning microscopy analyses.

### 3.5. RNA-Seq Data Processing and Analysis

Utilizing the Illumina sequencing platform, 25,902,410 raw reads were collected for the BB-11 control group, with 25,820,182 clean reads (99.68%) obtained after strict filtration; the BA-26 control group produced a total of 22,096,186 raw reads, and 22,015,042 (99.63%) clean reads were obtained after filtering. The BB-11 low-temperature treatment group produced 24,717,450 raw reads and 24,627,788 clean reads (99.63%); the BA-26 low-temperature treatment group produced 24,585,384 raw reads and 24,436,924 clean reads (99.39%). The Q20 values of the samples were >98%. After further removal of contamination and low-quality sequences, all remaining reads were aligned to the reference transcriptome to map to the existing gene annotations, which contained 2745 genes. A total of 2745 known genes were successfully annotated, and no new genes were identified. All these data indicated that the sequencing quality was sufficiently high for further analysis.

### 3.6. Differentially Expressed Genes (DGEs) in S. aureus with Different Responses to Low Temperature

**Figure 5:**
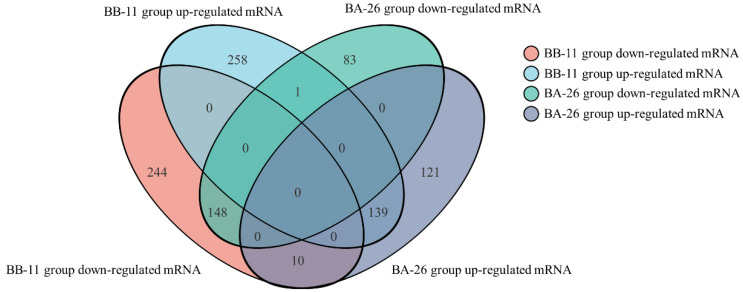
Distribution of differentially expressed genes (DEGs) between samples. DEGs were analyzed with the DEseq2 method, and DEGs were defined based on the following criteria: |log2 (fold change)| ≥ 1.00 and an adjusted *p* value ≤ 0.05.

Figure 5 shows the common and uniquely expressed genes in two different *S. aureus.* Eight hundred and thirty-three genes were significantly differentially expressed in the BB-11 group, of which 424 genes were upregulated and 409 genes were downregulated. Five hundred and twenty-seven genes were significantly differentially expressed in the BA-26 samples, of which 292 genes were upregulated and 235 genes were downregulated. Interestingly, ten genes were upregulated in the BA-26 group but were downregulated in the BB-11 group; only one gene was upregulated in the BB-11 group but downregulated in the BA-26 group.

### 3.7. GO Analysis

**Figure 6:**
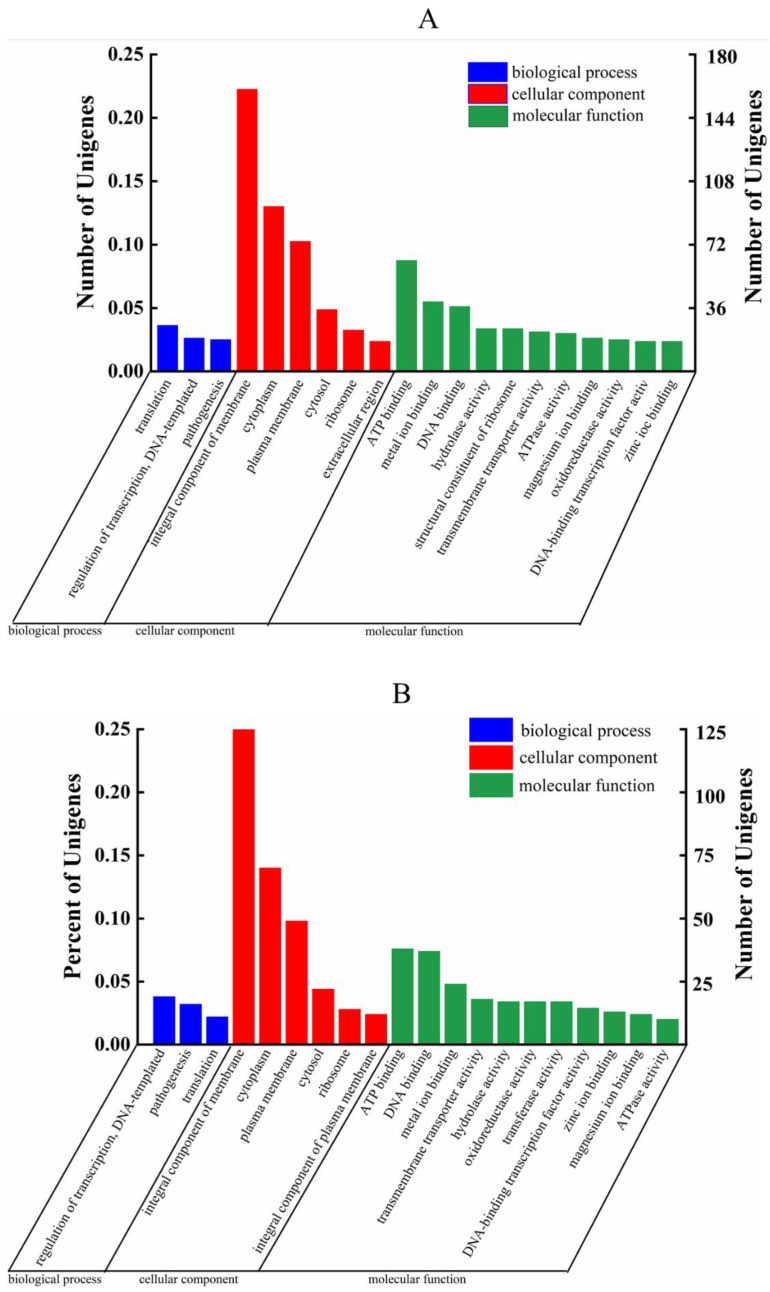
GO classification of DEGs in BB-11 (**A**) and BA-26 (**B**). The abscissa in the figure represents the secondary GO classification terms, the left ordinate represents the percentage of the total number of genes included in the secondary classification, the right ordinate represents the number of genes in the secondary classification in the comparison, and the three colors indicate the three main categories.

As shown in Figure 6, 70 DEGs were annotated in biological processes, 337 DEGs were annotated in molecular functions and 448 DEGs were annotated in cellular components in the comparison with the BB-11 group. In the comparison with the BA-26 group, 46 DEGs were annotated in biological processes, 219 DEGs were annotated in molecular functions and 294 DEGs were annotated in cellular components.

### 3.8. GO Enrichment Analysis and KEGG Enrichment Analysis

**Figure 7:**
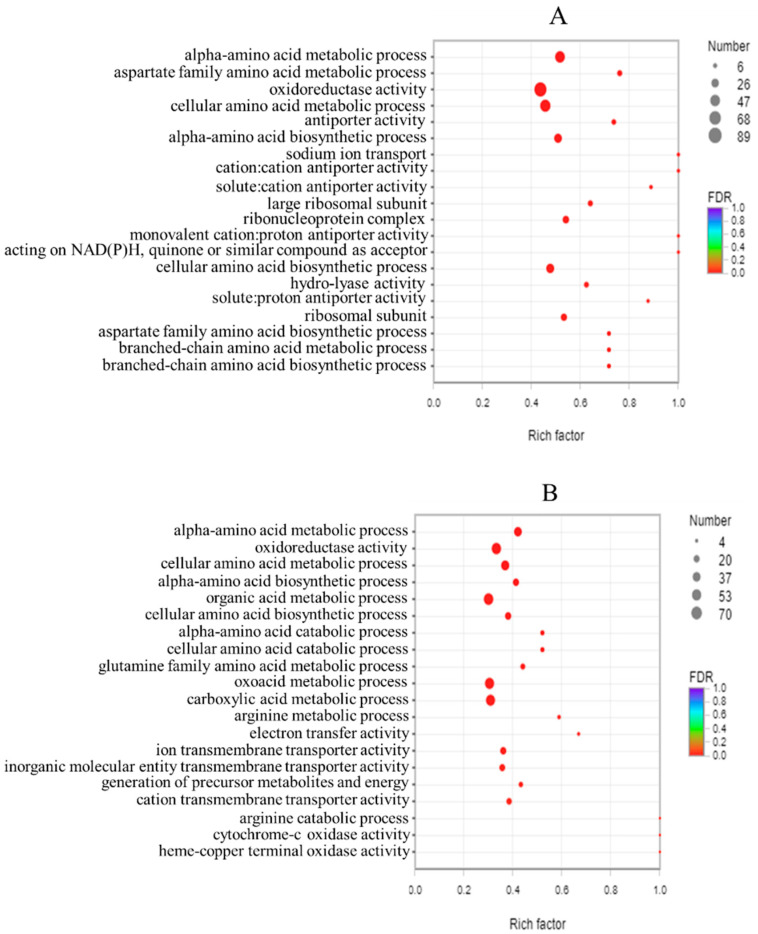
Scatter plots of the top 20 enriched GO terms for DEGs in BB-11 (**A**) and BA-26 (**B**). The vertical axis represents the GO term, and the horizontal axis represents the enrichment factor. The size of the dot indicates the number of genes/transcripts enriched in this GO term, and the color of the dot corresponds to different FDR ranges.

The top 20 functions annotated to enriched GO terms for the differentially expressed genes in two S. aureus strains are shown in Figure 7. The GO enrichment analysis mainly includes three major categories: biological process, cellular component, and molecular function. Sodium ion transport (GO: 0006814) was the dominant biological process observed in the BB-11 group. Cation: cation antiporter activity (GO: 0015491), monovalent cation: proton antiporter activity (GO: 0005451), and acting on NAD(P)H, quinone, or a similar compound as the acceptor (GO: 0016655) were the three dominant molecular functions identified in the BB-11 group. However, in the BA-26 group, two dominant molecular functions were identified, cytochrome-c oxidase activity (GO: 0004129) and heme-copper terminal oxidase activity (GO: 0015002), and one biological process was identified, the arginine catabolic process (GO: 0006527). The GO analysis showed that the DEGs were associated with various processes involving different molecular functions, biological processes, and cellular components.

**Figure 8:**
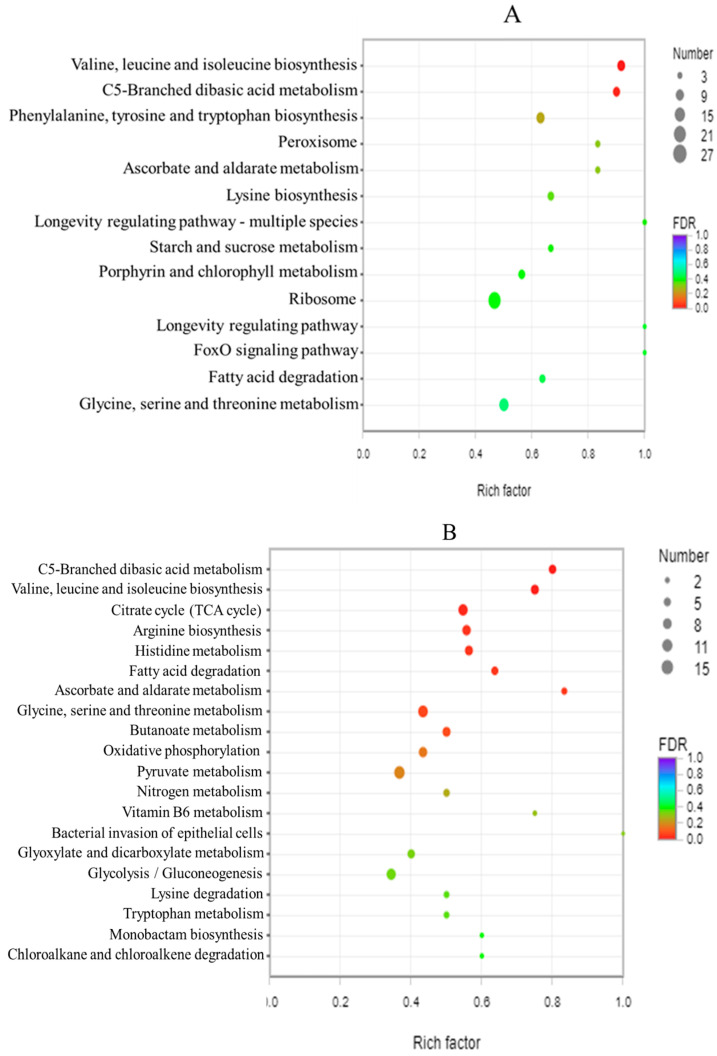
Scatter plots of enriched KEGG pathways for DEGs in BB-11 (**A**) and BA-26 (**B**). The vertical axis represents the name of the pathway, and the horizontal axis represents the enrichment factor. The size of the point represents the number of genes in this pathway in the gene set, and the color of the point corresponds to different ranges of FDR values.

In Figure 8, according to the KEGG pathway classification results, the most enriched pathways included the longevity regulating pathway (KEGG ID: map04213) and the FoxO signaling pathway (KEGG ID: map04013, KEGG ID: map04068, and KEGG ID: map05016) in the BB-11 group. In the BA-26 group, bacterial invasion of the epithelial cells (KEGG ID: map05100), ascorbate and aldarate metabolism (KEGG ID: map00053), and C5-branched dibasic acid metabolism (KEGG ID: map00660) were the main enriched pathways.

### 3.9. qRT–PCR Validation

**Figure 9:**
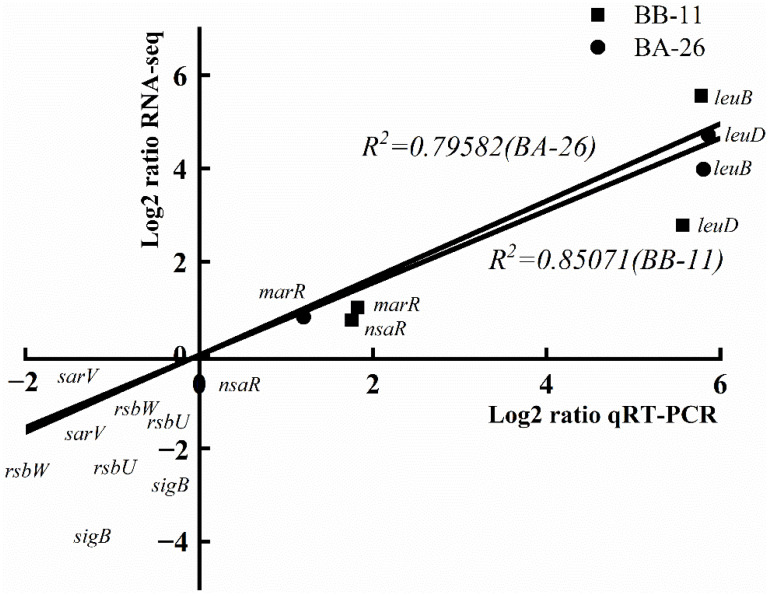
qRT–PCR validation. The *x*-axis indicates the log2 fold change obtained using RNA-seq analysis; the *y*-axis indicates the log2 fold change obtained using qRT–PCR.

Eight randomly selected genes were verified using qRT–PCR, and the results were compared with the results of RNA-seq to determine the consistency of the two approaches; the results are shown in Figure 9. Moreso, qRT–PCR data correlated well with the RNA-seq data (BB-11 group: *R*^2^ = 0.79582, BA-26 group: *R*^2^ = 0.85071). Generally, the qRT–PCR data were similar to the RNA-seq analysis of these genes, although the specific fold change values were different.

### 3.10. Differentially Expressed Genes (DEGs) in Response to Low-Temperature Treatment 

#### 3.10.1. DEGs Related to Fatty Acid Synthesis in The Cell Membrane of *S. aureus*

Based on the results of confocal laser scanning microscopy and transmission electron microscopy, the two strains of *S. aureus* exhibit quite different cell structures. The cell membrane integrity of BB-11 was better than BA-26. Fatty acids are the main components of cell membranes, and bacteria respond to environmental changes by changing the fatty acid composition of cell membranes mainly by altering the expression of related genes in the fatty acid synthase system. The expression levels of *fab* family genes related to fatty acid synthesis in the cell membrane were quite different between BB-11 and BA-26, as shown in Table 2. The expression level of the *fabG* gene in BB-11 was significantly higher than that in BA-26 (*p* < 0.05) at 1.056 and 0.46 log2(FC), respectively; however, the expression of the *fabZ* gene was downregulated in BB-11 and BA-26 by −2.53 and −0.82 log2(FC), respectively. 

**Figure 10:**
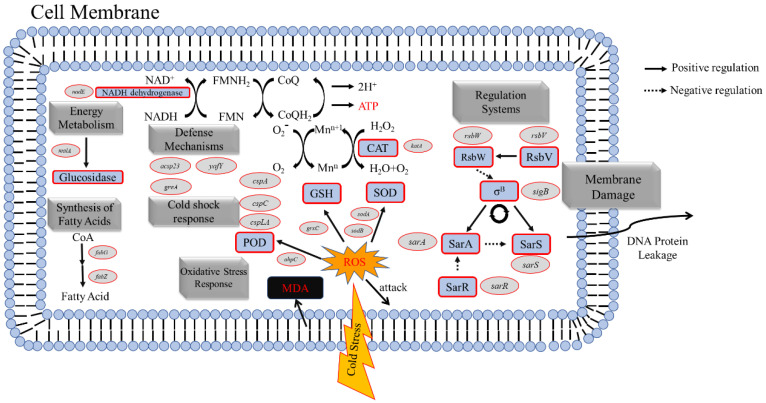
The response of *S. aureus* to a low temperature in vivo includes a cell structure response, oxidative stress response, energy metabolism response, regulation systems, and defense mechanisms. The oval box represents the DEGs, and the dotted arrows represent the various responses that are detrimental to the organism. Arrows indicated the activation, while the blunt-end arrows represented repression.

The *fab* family genes are mainly involved in regulating fatty acid synthesis in cell membranes (Figure 10); *fabG* encodes ketoreductase, which reduces ethyl4-chloro-3-oxobutanoate [31], and *fabZ* encodes an enoyl-acyl carrier protein reductase with a role in the fatty acid biosynthesis step and completion of the extension step [32]. Through RNA-seq analysis, we found that the transcription of *fab* family genes in the two strains of *S. aureus* was substantially altered after low-temperature treatment, and only *fabG* expression was upregulated in BB-11. The upregulation of *fabG* expression may contribute to the production and maintenance of cell membranes to provide a lipid supply, similar to the results of previous studies [10,33]. Therefore, low temperatures damage cells membranes, and the upregulation of the *fabG* gene in BB-11 may repair damaged cell membranes following exposure to a low temperature and resist low-temperature stress to improve the cell membrane structure of BB-11 compared with BA-26.

#### 3.10.2. DEGs Related to Oxidative Stress in *S. aureus*

After a long period of low-temperature treatment, the antioxidant system may be destroyed, causing an increase in ROS levels in the cell [34]. SOD is an important part of the microbial redox system [35] and an important ROS scavenger for the cell, which plays a key role in the cellular antioxidant system that protects the cell from damage. Reduced glutathione is a widespread antioxidant in organisms, which scavenges free radicals and protects the structure and function of cell membranes [36] Catalase metabolizes hydrogen peroxide into water and oxygen to prevent oxidative damage to cells [37], and increased levels of catalase will reduce lipid peroxidation [38]. Peroxidase is also an oxidoreductase that converts ROS into less harmful products to reduce the damaging effect of hydrogen peroxide on cells [39]. However, MDA is one of the peroxidation products of bacterial lipid membranes [40]—when the oxidative stress response of bacteria is disrupted, the danger of ROS produced by the organism attacking cell membranes is intensified, thus leading to an increase in the lipid peroxide MDA content of *S. aureus*. The combined actions of these enzymes and antioxidants may constitute the body’s protective mechanism against oxidative stress, which removes excess ROS.

In the analysis of the oxidative stress response (Figure 10) (Table 2), the *sodA* and *sodB* genes encoding SOD and Mn/Fe-SOD, respectively, were downregulated in BB-11 and BA-26. The genes (*katA*, *ahpC* and *gexC*) encoding catalase, peroxidase, and glutathione were upregulated 1.19, 1.02 and 1.35 log2(FC), respectively, but were downregulated by 0.71, 0.24 and 0.24 log2(FC) in BA-26, respectively. 

Because the low-temperature environment is not the optimal temperature for enzymes in microorganisms, the SOD activity of the two strains showed a decreasing trend. The significant increase in the reduced glutathione content in BB-11 enabled it to scavenge ROS and maintain the integrity of cell membranes. The SOD gene expression level verified the results of the preliminary measurement of SOD activity, and the downregulation of the *sodA* and *sodB* genes decreased the SOD enzyme activity in *S. aureus* under low-temperature treatment. Although BB-11 related genes were downregulated, their enzyme activity was higher than that in BA-26. A potential explanation for this finding is the improved cell membrane integrity that provides a stable internal environment suitable for the activation of the relevant oxidative stress response. With the increase in the low-temperature treatment time, the SOD activity in *S. aureus* gradually decreased, which might lead to the accumulation of ROS attacking fatty acids in the cell membrane to produce an increase in the MDA content [33]. However, the SOD activity and reduced glutathione content in BB-11 were higher than those in BA-26, and the MDA content was less than that in BA-26. The results were consistent with the RNA-seq data showing that the genes encoding catalase and peroxidase were upregulated, suggesting that changes in the levels of related genes contributed to the stronger oxidative stress defense system in BB-11 than that in BA-26 at low temperatures.

#### 3.10.3. DEGs Related to Energy Metabolism in *S. aureus*

Among the genes that encode proteins involved in energy metabolism, the NADH dehydrogenase gene (*nadE*) was upregulated 2.59 log2(FC) in BB-11 compared to BA-26; the *malA* gene encoding α-glucosidase was upregulated 3.34 log2(FC) in BB-11 but was downregulated −1.39 log2(FC) in BA-26. NADH dehydrogenase is the largest bacterial electron transport complex that transfers electrons directly to the respiratory chain through redox reactions and generates energy for use in cellular processes [41]. Moreso, α-Glucosidase is an exoenzyme widely found in bacteria, and its mode of action is similar to that of glucoamylase on disaccharides, oligosaccharides, and aryl glycosides and produces glucose [42]. According to a previous study, when the cellular energy metabolism system is destroyed, the metabolic rate is decreased, systematic disorders occur and cell survival is affected [43]. Therefore, strain BB-11 may upregulate the expression of genes encoding NADH dehydrogenase and α-glucosidase, stabilize energy metabolism and allow other systems to operate normally. The downregulated expression of the genes encoding NADH dehydrogenase and α-glucosidase in the strain BA-26 may disrupt energy metabolism and induce cell death.

#### 3.10.4. DEGs Related to The Regulatory System of *S. aureus*

*S. aureus* senses different environmental factors and adjusts its response to these environmental signals [44]. The sigB (σ^B^) factor is one of the important factors regulating the environmental stress response in bacteria, which is encoded by the *sigB* gene. The regulation of the *σ*^B^ factor activity is achieved through a “partner-switching mechanism” [45]: when the organism is in a stress-free environment, the RsbW protein (anti-σ^B^ factor, encoded by the *rsbW* gene) phosphorylates the RsbV protein (anti-anti-σ^B^ factor, encoded by the *rsbV* gene), disrupting the binding of RsbV to RsbW, blocking the antagonism of the RsbW protein, and allowing the σ^B^ factor to bind to the RsbW protein and inhibit its activity; when the cell is under stress, RsbV is dephosphorylated and binds to RsbW, causing the release of σ^B^ to induce a transcription of genes that rely on the σ^B^ factor.

The regulation of SarA, another important transcription factor, depends on the control of the σ^B^ factor, which regulates mRNA lifetime at the posttranscriptional level to regulate the expression of target genes [46]. Another protein, SarR, with a 51% similarity to the SarA factor inhibits the transcription of the SarA factor [47]. SarA factor negatively regulates SarS protein. SarS protein is a positive regulator of *spa* transcription, but it is activated by ClpXP protease [48]. The experimental data showed that the expression levels of the *sigB* gene, *rsbW* gene, and *rsbV* gene were significantly reduced after 1 week of low-temperature treatment in both strains of *S. aureus*, but the expression levels of the *sarA* gene were significantly upregulated in both strains, indicating that the SarA factor plays a positive role in the resistance of *S. aureus* to low temperatures.

#### 3.10.5. DEGs Related to The Defense Systems of *S. aureus*

A stress protein encoded by the *asp23* gene is associated with the adaptation of bacterial strains to harsh environments, and deleting the gene from the genome increases the sensitivity of *S. aureus* [49]. Notably, *greA* encodes the transcription elongation factor GreA that affects bacterial gene transcription by regulating gene promoters, thereby regulating bacterial environmental adaptation [50]. The endosomal lipoprotein YafY, which is encoded by *yafY*, strongly induces *degP* expression, the gene encoding periplasmic protease that is thought to be required for growth under adverse conditions [51]. The RNA-seq data showed that the expression of the *asp23*, *greA,* and *yafY* genes was upregulated 1.02, 1.49, and 1.43 log2(FC), respectively, in BB-11, but were downregulated 0.25, 0.53, and 0.71 log2(FC), respectively, in BA-26. Thus, when BB-11 is located in an unfavorable environment, the cells are protected from external stress by the response of defense systems within the organism, which was elucidated years before by other researchers [49]. Therefore, these genes played an active role in the resistance of strain BB-11 to low-temperature treatment, while these genes did not seem to be helpful in BA-26.

#### 3.10.6. DEGs Related to Cold Shock Stress in *S. aureus*

After 1 week of low-temperature treatment, both strains of *S.* *aureus* exhibited significantly reduced expression of cold-shock proteins. Cold-shock proteins are part of the survival mechanism of microorganisms to adapt to the low-temperature environment, and cold shock reactions have been identified in many microorganisms [15]. *CspA* was the first cold-shock protein to be discovered, and it increases the expression of proteins related to low-temperature adaptation [52]. An increasing number of research results show that cold-shock proteins are not only involved in the host’s cold shock response but are also necessary for the normal growth of the host. *CspC* participates in the nutritional starvation stress response of *S. aureus* and is related to the activity of stable cells [53]. *CspLA* also protects DNA from damage [6]. Studies have shown that short-term low-temperature treatment upregulates the expression of *csp* family genes [54]. Our RNA-seq data showed that the expression of *csp* family genes was downregulated in both *S. aureus* strains after long-term low-temperature treatment. Nonetheless, *csp* family genes were downregulated to a greater extent in BA-26 with a weak low-temperature resistance and downregulated to a lesser extent in BB-11 with strong low-temperature resistance. These proteins may be part of an immediate response to cold shock, and once the bacteria are acclimated, these proteins are homeostatically unrelated to 1 week of low-temperature treatment.

## 4. Conclusions

This study systematically compared the differences in cell surface morphology, intracellular enzyme activity, and gene expression between two strains of *S. aureus* exposed to low temperatures. After low-temperature treatment, the low-temperature survival of BB-11 is better than that of BA-26, and BB-11 also shows a better cell membrane integrity and in vivo oxidative stress response than BA-26. RNA-seq data verify the upregulation of the expression of cell membrane fatty acid-related genes and oxidative stress-related genes, confirming that the cell membrane integrity and the oxidative stress response of BB-11 were better than those of BA-26. Additionally, energy metabolism and the defense system in BB-11 are also actively involved in the resistance to low temperatures. Therefore, these molecular mechanisms together result in the better survival of BB-11 at low temperatures than BA-26. This study sounds an alarm for the risks posed by stress tolerance of *S. aureus* in the frozen food industry.

## Figures and Tables

**Table 1:** Primers used for qRT–PCR.

Genes	Primer	Sequence (5′-3′)	Product Length (bp)	References
*leuB*	*leuB-F*	GGTGCAATCGGTGGACCTAA	124	this research
	*leuB-R*	TAGCGCCTTTGACAACGGTA		
*leuD*	*leuD-F*	TGGAAACGCTTGTCTGGTGA	211	this research
	*leuD-R*	GTCGTGAACATGCTGCTTGG		
*sigB*	*sigB-F*	TGGAGTGTACATGTTCCGAGAC	75	this research
	*sigB-R*	AGCGGTTAGTTCATCGCTCA		
*sarV*	*sarV-F*	TCATCCGTTTCAGAACGCAA	141	this research
	*sarV-R*	TGAAGCTGAAAAGATTAGCGGT		
*rsbW*	*rsbW-F*	GCGAAGGTGGCCTAGGTTTA	125	this research
	*rsbW-R*	GCCATTATTTCGCACCTGCT		
*NsaR*	*NsaR-F*	GCGCGTCATGTTAACAGCTA	273	this research
	*NsaR-R*	ACGATTGCCAAAATTCAAGCAT		
*MarR*	*MarR-F*	AATAAGGCCGCAGTAAGCCG	166	this research
	*MarR-R*	GCGCAATATCTGTCATAATCGCA		
*rsbU*	*rsbU-F*	GCTGGTCATGAGCCTGGATA	171	this research
	*rsbU-R*	AGCTTCAGTCACACCATCCG		
*16s rDNA*	*16s rDNA-F*	CGGTGAATACGTTCYCGG	124	[17]
	*16s rDNA-R*	GGWTACCTTGTTACGACTT		

**Table 2:** Classification of DEGs of *S. aureus* involved in the response to cold temperature.

Gene ID	Symbol	*S. aureus* BB-11	*p*-Value	*S. aureus* BA-26	*p*-Value	Description
		Fold Change/Log2(FC)		Fold Change/Log2(FC)		
*Fatty acid biosynthesis in membrane*						
E3306_06220	*fabG*	1.056	0.001266	0.46	0.032878	3-oxoacyl-[acyl-carrier-protein] reductase
E3306_11095	*fabZ*	−2.53	4.61 × 10^−13^	−0.82	0.001912	3-hydroxyacyl-ACP dehydratase FabZ
*Oxidative stress*						
E3306_08020	*sodA*	−1.55	3.61 × 10^−6^	−0.66	0.002897	Superoxide dismutase
E3306_00485	*sodB*	−1.52	1.07 × 10^−5^	−0.73	0.003111	[Mn/Fe]-Superoxide dismutase
E3306_06825	*katA*	1.19	3.74 × 10^−4^	0.71	0.004024	Catalase
E3306_13890	*grxC*	1.35	3.60 × 10^−4^	0.24	0.399629	Reduced Glutathione
E3306_01835	*ahpC*	1.02	0.011891	0.24	0.223756	Peroxidase
*Cold shock response*						
E3306_07190	*cspA*	−1.78	3.08 × 10^−8^	−1.25	5.58 × 10^−6^	Cold-shock protein (CspA)
E3306_14325	*cspLA*	−1.21	0.063056	−3.24	6.03 × 10^−23^	Cold-shock protein (CspLA)
E3306_04085	*cspC*	−0.66	0.284248	−2.27	8.17 × 10^−18^	Cold-shock protein (CspC)
*Energy Metabolism*						
E3306_02110	*nadE*	5.33	4.47 × 10^−64^	2.74	1.68 × 10^−9^	NADH dehydrogenase
E3306_07745	*malA*	3.34	2.21 × 10^−21^	−1.39	1.37 × 10^−5^	α-glucosidase
*Regulation System*						
E3306_10900	*sigB*	−1.57	2.20 × 10^−8^	−0.65	0.001570	RNA polymerase sigma factor SigB
E3306_10910	*rsbV*	−1.86	2.20 × 10^−12^	−0.38	0.120798	STAS domain-containing protein(RsbV)
E3306_10905	*rsbW*	−1.64	2.42 × 10^−7^	−1.05	0.006128	anti-sigma B factor RsbW
E3306_03140	*sarA*	2.89	2.40 × 10^−19^	2.44	6.38 × 10^−6^	transcriptional regulator(SarA)
E3306_00370	*sarS*	−2.81	2.99 × 10^−20^	−1.83	9.22 × 10^−4^	HTH-type transcriptional regulator SarS
E3306_12130	*sarR*	0.24	0.675713	−0.49	0.071711	HTH-type transcriptional regulator SarR
*Defense Mechanism*						
E3306_07880	*asp23*	1.02	1.26 × 10^−4^	0.25	0.221559	Asp23/Gls24 family envelope stress response protein
E3306_08310	*greA*	1.49	1.03 × 10^−9^	0.53	0.048271	transcription elongation factor GreA
E3306_12230	*yafY*	1.43	6.30 × 10^−5^	0.71	9.07 × 10^−5^	YafY family transcriptional regulator

## Data Availability

The data presented in this study are openly available in the Sequence Read Archive (SRA) of the National Center for Biotechnology Information Search (NCBI) database under accession code PRJNA818660.
